# “I felt like a TRIO champion”: end-user perspectives on their role as co-designers of multi-purpose technologies

**DOI:** 10.12688/gatesopenres.13182.1

**Published:** 2020-10-26

**Authors:** Kawango Agot, Alexandra Lutnick, Mary Kate Shapley-Quinn, Khatija Ahmed, Timothy Okello, Ariane van der Straten, The TRIO Study Team

**Affiliations:** 1 Impact Research and Development Organization, Kisumu, Kenya; 2 Behavioral and Urban Health Program, RTI International, San Francisco, California, USA; 3 Women's Global Health Imperative, RTI International, Berkeley, California, USA; 4 Setshaba Research Centre, Soshanguve, Gauteng, South Africa; 5 Department of Medicine, Center for AIDS Prevention Studies, UCSF, San Francisco, California, USA

**Keywords:** Co-designer, multipurpose technologies, end-users, HIV prevention, contraception, women, Africa, PrEP

## Abstract

**Background:** The likelihood that research will be relevant to and accepted by end-users and their communities is enhanced when the perspectives of both the “researchers” and the “researched” are considered. The Tablets, Ring, Injections as Options (TRIO) Study, conducted with young women in Kenya and South Africa, assessed the acceptability and preferences of three placebo-only multi-purpose technology (MPT) forms for prevention of HIV and unintended pregnancy. The objective of this analysis was to assess whether, and if so how, the women participating in the TRIO Study perceived themselves as co-designers of the three MPT products.

**Methods:** We conducted 55 in-depth interviews, 6 focus group discussions, and 5 dissemination workshops with TRIO Study participants. Woven throughout these activities were questions and opportunities for participants to reflect on their role in the study, and to what extent they identified with their role as a co-designer. Qualitative data from these activities were analyzed thematically.

**Results:** The analysis revealed four key themes about what resulted in the women’s views as co-designers: altruism, respectful treatment, agency, and reciprocity. The women were aware of their role in determining what end-users would and would not prefer and were motivated by a desire to help themselves and others. They recognized their role as co-designers and cited being treated well by study staff, being given a chance to make choices during the study period, and being recognized as equal partners of the researchers as the main reasons.

**Conclusions:** If prevention products are going to be successfully developed, end-users and researchers must work hand in hand. Engaging participants meaningfully as co-designers in product development research can be a powerful tool in the effort to ensure new prevention products brought to market are acceptable to the population of interest.

## Introduction

In sub-Saharan Africa, adolescents and young people aged 15–24 years make up only 10% of the population yet accounted for 32% of new HIV infections in 2019 ( UNAIDS, 2020). Adolescent girls and young women in the sub-continent are more disproportionately affected, accounting for 62.5% of the new infections among adolescents and young people ( UNAIDS, 2020) and acquiring HIV 5 to 7 years earlier than their male counterparts ( Dellar *et al.*, 2015). Despite the increased vulnerability of adolescent girls and young women to HIV in sub-Saharan Africa, their uptake, retention, and adherence to HIV prevention interventions that work (notably pre-exposure prophylaxis), have been sub-optimal both during trials and rollout ( Celum *et al.*, 2019; Koss *et al.*, 2020; Were *et al.*, 2020). It is hypothesized that, when new products are discussed with future end-users before development and their opinions are considered at the design stage, uptake and adherence are likely to improve ( Guthrie *et al.*, 2018; Shah & Robinson, 2007; Swain *et al.*, 2019). Therefore, end-user input is increasingly considered essential for product development to ensure their attributes and usage preferences help guide the development process ( Brady & Tolley, 2014; Guthrie *et al.*, 2018; Thilo *et al.*, 2017).

Most end-user studies focus on bringing together potential users and researchers/programmers to co-design strategies for roll-out after the intervention/product has been developed ( Slater *et al.*, 2017). At this point it may be too late or too expensive to consider modifying product attributes that may be unacceptable to end-users. The likelihood that research results will be relevant to and valued by end-users and their communities is enhanced when the perspectives of the “researchers” and the “researched” jointly inform the design of the intervention products ( Montgomery *et al.*, 2017). It is therefore prudent to bring end-users on board earlier in the product design stage to partner with developers and researchers in designing products that would be more acceptable — and adhered to when put on the market ( Krogstad *et al.*, 2018; Montgomery *et al.*, 2017). The co-designer concept, in which the three key players (developers, researchers, and end-users) collaborate, encourages end-users to take an equal role in the design of healthcare interventions ( Bate & Robert, 2006).

We conducted a study to assess the acceptability of and preferences for the attributes and routes of administration of three placebo-only multi-purpose technology (MPT) products (tablets, ring, and injections) for prevention of HIV and unintended pregnancy among young women in Kenya and South Africa ( van der Straten *et al.*, 2018). As part of joining the study, participants were invited to engage as co-designers so that their feedback could help researchers and product developers improve the products they would receive and use. The purpose of the sub-analysis informing this paper was to assess whether, and if so how, the young women who participated in the study perceived themselves as co-designers in the development of the three MPT products being investigated.

## Methods

Tablets, Ring, Injections as Options (TRIO) was a 5-month clinical study conducted at the Impact Research and Development Organization (IRDO) in Kisumu, Kenya and Setshaba Research Centre (SRC) in Soshanguve, South Africa. Study enrollment occurred between December 2015 and June 2016, with follow-up visits completed in December 2016 and dissemination of results in May/June 2017. Details of the methods have been presented elsewhere ( Minnis *et al.*, 2018; van der Straten *et al.*, 2018). Briefly, women were eligible to participate if they were aged 18 to 30 years, sexually active, not pregnant, HIV negative, and had no prior participation in HIV prevention product trials or demonstration studies. The three placebo-only MPTs that the women used and assessed were daily oral tablets, a monthly silicone vaginal ring, and two 2 mL monthly saline injections. This analysis focuses on findings from the qualitative data collected throughout the study, including during dissemination of results, to explore participants’ perceptions of being invited to engage as co-designers in the development of the MPTs.

Strategies for engaging TRIO participants started during recruitment and continued through to the end of the study ( Table 1). Pre-enrollment workshops were held to introduce interested community members to the study and the placebo products being evaluated and to answer any questions. Once enrolled in the study, women received telephone check-ins between their monthly visits. Participants received a certificate at their month 5 visit with stars for each time they gave a pledge to provide honest responses about what they really thought of the products during monthly interviews. Additionally, suggestion boxes were available at the clinic for those wanting to provide anonymous feedback about their study experiences, and “Chill sessions” (where participants came to the study site to simply hang out) were conducted (at SRC site only) so that participants could receive more education about the study and to facilitate an atmosphere of trust, openness, and partnership among participants and the research team.

**Table 1.** Engagement activities by site and overall for TRIO.

	IRDO	SRC	Total
Pre-enrollment workshops			
No. Conducted	13	33	44
No. of workshop participants	387	289	676
No. Phone check ins/No. participants contacted	358/126	347/135	705/261
No. Certificates of completion provided	122	124	246

IRDO, Impact Research and Development Organization; SRC, Setshaba Research Centre; TRIO, Tablets, Ring, Injections as Options Study.

The qualitative data sources ( Table 2) used for this analysis include 55 in-depth interviews (IDIs), six focus group discussions (FGDs), and five dissemination workshops, all conducted at the research sites. IDIs were conducted with randomly selected participants following their month 3 visit in TRIO, or with a purposive sample of women based on their decision to switch products during the usage period. The 37 women who participated in the six FGDs at study exit were purposively selected and stratified according to the product selected during the usage period. Detailed methods related to the IDI and FGD component are previously reported elsewhere ( Shapley-Quinn *et al.*, 2019).

**Table 2.** Qualitative activities by site and overall for the TRIO qualitative sample.

	IRDO (N=90)	SRC (N=75)	Total (N=165)
No. IDIs	25	30	55
No. FGDs (No. participants)	3 (n=18)	3 (n=19)	6 (n=37)
No. Dissemination workshops (No. of participants who joined)	2 (n=71)	3 (n=46)	5 (n= 117)

Forty participants completed either an IDI or FGD, and a dissemination workshop; three completed an IDI, FGD, and attended a dissemination workshop. FGD, focus group discussion; IDI, in-depth interview; IRDO, Impact Research and Development Organization; SRC, Setshaba Research Centre; TRIO, Tablets, Ring, Injections as Options Study.

Approximately 6 months after the study ended, we conducted dissemination workshops at each site with a total of 117 former study participants to share the key findings and solicit the women’s feedback about those findings; the results have been presented elsewhere ( Agot *et al.*, 2019). The workshops also explored how participants viewed their role as co-designers during their participation in TRIO and heard their recommendations to enhance a co-designer relationship between researchers and participants in future studies (see extended data for slide ( Wagner & Shapley-Quinn, 2020)).

All qualitative data collection was conducted by trained interviewers and facilitators in local languages. IDIs and FGDs were conducted using semi-structured guides (see extended data ( Wagner & Shapley-Quinn, 2020)), audio-recorded, transcribed, and translated into English when needed. The dissemination workshops were conducted using a structured guide. Dissemination workshops at SRC were audio-recorded, transcribed, and translated into English where necessary. A secure File Transfer Protocol site was used to transmit all audio recordings. Audio recordings were stored securely as protected health data until analyses were completed and audio recordings destroyed. At IRDO, three note-takers attended each workshop because the site did not apply for ethics approval to audio-record the workshop proceedings. Once the workshops were complete, the note-takers compared and triangulated their notes to develop full text.

### Data analysis

A codebook was developed for the IDI and FGD data, and three analysts coded all transcripts using Dedoose, a web-based qualitative analysis software (see extended data ( Wagner & Shapley-Quinn, 2020)). The average coder inter-rater reliability score was 0.82 (calculated as a pooled Cohen’s kappa). After coding was completed, code reports were generated for the following codes: “engagement,” “research,” “visits and procedures,” “TRIO Study,” and “recommendations,” and summary memos were written for each of these code reports. Thematic analysis was used with the transcripts (SRC) and expanded notes (IRDO) from the dissemination workshops to explore the contents of the discussions related to people’s perceptions of their role as co-designers.

### Ethics

The study obtained ethics approval from Scientific and Ethics Review Unit of the Kenya Medical Research Institute (Ref #: NON-SCC 474) and from South Africa's Pharma Ethics (Pty) Ltd (Ref #: 150110905). RTI International's IRB transferred oversight responsibilities to the Kenyan and South African IRBs through formal authorization agreements. The study was not registered with a clinical trials site as the products were all placebo.

## Results

Across the two TRIO sites 165 women participated in at least one qualitative activity. Over half of the women were ages 18–24 and had completed secondary school (see Table 3). Among those who joined one or more qualitative activities, four key themes emerged from the data about what resulted in the women feeling like co-designers; these included altruism, respect, agency, and reciprocity.

**Table 3.** Background characteristics of TRIO participants.

Characteristics	Total Sample		Qualitative sample *	
	N	(%)	N	(%)
	277	(100)	165	(100)
Age				
18 to 24	183	(66)	109	(66)
25 to 30	94	(34)	56	(34)
Education				
Completed secondary school	143	(52)	85	(52)
Income				
Earns an income	86	(31)	56	(34)
Relationship Status				
Currently has a primary partner	261	(94)	158	(96)
Married or cohabiting	79	(29)	53	(32)
Currently has a casual sex partner	50	(18)	27	(16)
Site				
IRDO	137	(49)	90	(55)
SRC	140	(51)	75	(45)

*Qualitative sample is comprised of women who participated in an IDI, FGD, and/or dissemination workshop. Forty participants completed either an IDI or FGD and also a dissemination workshop; three completed an IDI, FGD, and attended a dissemination workshop. FGD, focus group discussion; IDI, in-depth interview; IRDO, Impact Research and Development Organization; SRC, Setshaba Research Centre; TRIO, Tablets, Ring, Injections as Options Study.

### Altruism

Although women reported feeling special because they were *“among the few selected…to join the TRIO Study”* (IDI, IRDO), it was more important to many that they were representing and ultimately helping other women. This sense of service to others was noted by the women as a crucial aspect of their co-designer role. The women commented about how their involvement would save other women. During an individual interview at IRDO, one woman reflected that, *“I strongly felt that I should take the responsibility to save other women around the world if I participated in the study.”* Similarly, several discussants who participated in the dissemination workshops at IRDO said that the term included on the TRIO Certificates of Completion, *“shujaa*, *”* (champions/heroes) resonated with them, *“because I have been able to help women in Kenya and South Africa through being part of the research of these products”* and because they volunteered *“to help other women like me.”* Women who participated in the FGDs at SRC reflected that they, and sometimes even their family members, felt proud of themselves because their contribution to the design of the MPTs was *“gonna bring a change in the world.”*

The women’s altruistic motivation as co-designers was also expressed by their understanding that they were working directly with the research team to help develop products that women will like and want to use. It appears that this motivation to co-design the MPTs encouraged the women to communicate honestly their opinions about the products. Knowing that they were participating to help *“the generation to come”* (FGD, SRC), they seemed more inclined to *“….give true feedback because it is going to help other women. So when I was given a product and returned* (to the site) *, I made sure I gave honest answers”* (FGD, IRDO). As one woman shared during a dissemination workshop in SRC, *“The information I gave was honest. It will be able to help other women because I’m sure that what I like is not only liked by me alone, and my health and safety is like everybody else’s.”*

### Respect

The interviews revealed that for participants, it was extremely important that the research team extended the same respect to them as they would to their research colleagues. Women noted that the calls they received from the study staff made them feel appreciated, special, cared for, and part of the team: *“I felt appreciated like, at least like I’ve been remembered and these people like they care about their participants”* (IDI, SRC). In IRDO, a FGD participant described how she *“….felt so good when I received the phone call to find out how I was fairing on with the ring and said to myself, ‘so these people care so much about me that even though they gave me this thing* [ring] *if I feel any discomfort I can easily go back to have it sorted out.’”* This air of respect, caring, and openness was noted by many women as a key component of feeling like co-designers—like they and the researchers were working together to identify attributes of the MPTs that would be acceptable to women. Women in SRC were particularly appreciative of the kindness with which staff treated them and the confidentiality the staff maintained about their discussions. As one woman shared during a dissemination workshop, “ *You would never find that when you leave the room you would find* [name of staff] *telling other people that this one has had a lot of sex with her partner during the week.”* Participants were therefore able to share freely how they felt the products needed to be fashioned to fit the expectations of end-users.

The respect the women felt they were shown by the study staff helped facilitate open communication about their opinions and preferences. During dissemination workshops in both sites, a number of participants said reminders about attending clinic visits by study staff and receiving follow-up phone calls to find out how they were doing, and to update them about the study, made them feel part of the game: " *I like the love from the people at* SRC *, ever smiling, and then when you were unable to come they would call and ask why you could not make it and then you would apologize and they would ask when will you would be able to come and when you are able to come, they won’t cast you aside because it’s been a while since you came in; no they give you attention and you also feel at ease....if you have a problem, they listen to your problem. That is what Setshaba did for me so yes, well done."* A FGD participant at SRC also captured this mood clearly: " *It* [phone check in] *is very important because it shows us that we are important. You are not taking us for granted, that we are just doing the study and that’s it. Like you do everything in your power to show us how important we are…..after I’ve come here for the second time I just experienced that wow, no, these people are not just caring about their research.”* These statements emphasized that the care and attention from study staff gave participants a sense of belonging that made them open up to sharing honest views about what they liked and did not like about the products.

Understanding the ways in which women felt that the respect they were shown fostered the co-designer relationship; it is not surprising then that women who did not feel respected did not feel like they were important or part of the team. Some women felt disrespected because they had to endure long wait times at the clinic, they sometimes were not informed about what would take place during a study visit, or they felt the study team did not trust them. Explaining this lack of trust, one woman in an IDI at IRDO described that the study procedure around ring insertion and removal communicated that the staff did not believe the women would follow the directions: *“If you did not want to participate in the TRIO project, you would have refused and left it. Which means you accepted and agreed to do those things and that is the reason you accepted to be coming back, so I always don’t see the need that they insist you have to put in the ring here and you come and remove it here.”*

### Agency

For some, the study design itself awakened their perception of being co-designers. They described that because they were given the opportunity to choose a product to use during the usage stage of TRIO, they felt like they were *“study partners and not participants”* and that they were *“part of the team”* (dissemination workshop, IRDO). Putting this choice in their hands sufficiently disrupted the traditional top-down approach of research and resulted in the women feeling empowered and engaged. *“I felt I was involved because at some point I was the one who chose whatever I wanted, like it was up to me…I was able to say what was on my mind”* (IDI, SRC). Recognizing their agency, the women reflected that because they are co-designers, the *“top dogs”* (i.e., “big” people [important people, decision makers] with the money who come up with new things) valued their opinions as *“professionals”* and that *“in the end they’ll take my opinion on whatever I tell them”* (IDI, SRC). The women understood that without them *“there won’t be any survey conducted”* (IDI, SRC). **

### Reciprocity

In their role as co-designers, the women spoke about the mutually beneficial nature of their relationship with the research team. In addition to the remuneration they received for their participation, several women also reported that they gained knowledge and skills as co-designers. A participant at a dissemination workshop in IRDO shared that *“I did not know that there could be a product that prevents both pregnancy and HIV, but now I have knowledge and experience and I feel good.”* For others, their involvement changed their perceptions about the types of prevention products they would be willing to use. One of the participants during an IDI at SRC explained, *“I’ve never thought that I’ll—even a female condom, I never thought I’ll use it. Like putting something in my vagina. So when I got there, I changed my mind, my mindset* (on) *how things are done. I was willing to do it and it was quite an experience for me.”* Feeling empowered to co-design the products both for their own benefit and for the benefit of others was also expressed by a FGD participant at IRDO: “ *I can now teach somebody else who is ignorant on HIV and family planning issues the way I have been taught and make them understand with the hope that suppose that the medicine will be there* (when the MPT products will be active, not placebo) *, then I am in a position to influence a fellow woman to access it and get helped*.” Knowing such technical topics resulted in some women feeling elevated to the level of a community resource person.

To demonstrate the reciprocal nature of the TRIO Study further, the research team provided Certificates of Completion. In both IDIs and FGDs women discussed how the certificates made them feel important, that they were a part of positive change for their communities, and they were able to see something through to completion. During a FGD at SRC, a woman shared how it *“*…. *made me feel proud of myself because I was able to begin something and go through to the ending.”* Some thought that they may even be able to include the certificate in future job applications as evidence of their ability to complete significant tasks and that they engaged in *“voluntary work”* (IDI, SRC). Lastly, one woman spoke about how meaningful it was to her that she received the certificate, especially as someone who had not completed high school or college. She said, *“The certificates, it felt, it felt so good because some of us never got the chance to go to tertiary…So receiving the certificate meant a lot you see…more than the Malu –Malusi* [matric certificate] *you see. So yes, it felt good indeed”* (FGD, SRC).

## Discussion

Although the main purpose of the TRIO Study was to obtain end-user perspectives into the development of three potential MPT products—tablets, ring and injections, the objective of this analysis is to present how participants perceived their role as co-designers of the three products being evaluated. Trying out each product and sharing their views on the attributes they liked or did not like, then making suggestions on aspects that could be modified made participants view themselves as more than a mere source of information for researchers; they felt like co-creators of the products. Even though the products are already designed and at different stages of clinical development as HIV prevention methods, none existed yet as MPTs or dual prevention products for pregnancy and HIV prevention. Furthermore, participants’ co-designer ideas truly contributed to identifying barriers to each of the product forms as well as potential solutions. As reported in another paper from the TRIO Study ( Minnis *et al.*, Under review), participants came with suggestions for improved product dosage and delivery modalities currently being evaluated by developers. These included a weekly pill ( Kirtane *et al.*, 2018), a transdermal patch ( Puri *et al.*, 2019; Vogler *et al.*, 2010), and longer acting bi-monthly injections ( Kerrigan *et al.*, 2020). Thus, involving end-users early in product development can inform product attributes before form factors are locked in ( Krogstad *et al.*, 2018; van Velsen *et al.*, 2018).

In a systematic review of 49 studies addressing the development of supportive technologies for persons with dementia, Suijkerbuijk *et al.* (2019) explored the level of engagement with end-users as co-designers, driving the process into four phases: pre-design (exploring people’s lived experiences that would make a product necessary), generative (exploring ideas of the product design), evaluation (iterative development and testing of the product in different formats with representative end-users), and post-design (actual use of the product while also monitoring acceptability). The authors reported that only 7 of 49 studies included people with dementia in multiple phases of development and iteratively consulted them across all the four co-designer phases while, similar to our study, the majority engaged end-users in the development (38/49) and generative (25/49) phases only. Importantly though, 38 (77.6%) of the studies reported explicitly that the recommendations by the end-users resulted in one or more changes in the design of the final product (11 studies did not report on this).

It was important for us to find out whether, in taking part in the study, participants were aware that they were co-designers of these products and that their opinions would be considered by product developers in designing new biomedical interventions. This is important in two ways: one, participants would appreciate the important role they play in determining how the products would look like when released into the market for use and two, in knowing and appreciating their co-designer role they would better understand the importance of providing their honest views.

As experts of their own lives, end-users should be offered an opportunity to influence the design processes of products being development for them ( Giroux *et al.*, 2019; Hussey *et al.*, 2019), and working collaboratively can optimize the eventual acceptability of the interventions. Results from our study indicate that the women appreciated the respect accorded to them and the recognition that they as end-users and researchers must work hand in hand for the successful development of prevention products. Latkin and colleagues ( Latkin *et al.*, 2016) noted that a key to overcoming social desirability within research is to treat participants with respect, a factor also considered important by our participants. Participants were clearly aware of their role in determining what the end-users would and would not prefer. For some, volunteering to participate in the research to help other women made them feel like heroes. In joining the TRIO Study to share their experiences and preferences of the three dual-use products, participants made the statement that they were volunteering for the benefit of others. Joining a study for altruistic reasons has been expressed by participants in other studies, including one that examined acceptability of vaginal ring for HIV prevention ( Montgomery *et al.*, 2017).

Altruism alone is not enough to maintain a co-designer relationship. In co-designing health products, future end-users are empowered through being engaged as experts of their experiences ( Cottam & Leadbeater, 2004; Taffe, 2015; Wetter-Edman, 2012). True to a participatory approach to the research process, the TRIO Study provided the women with new information on a topic of concern to them, worked with the women to increase the resources available to their community, delivered the study findings back to them and asked for their perceptions on those results, and translated what was learned from the women into strategies that will hopefully meet their HIV and unintended pregnancy prevention needs. Ideas from potential end-users better match the needs of the actual end-user than the ideas generated by professional developers or researchers ( Kristensson & Magnusson, 2010). By engaging local knowledge, the quality and validity of the research is improved ( Hussey *et al.*, 2019). Thus, it is important to recognize the symbiotic relationship between product developers who understand what is technologically feasible and end-users who experience the product and therefore know what is best for them.

This study has several limitations to consider. The women who participated in IDIs, FGDs, and/or dissemination workshops may not represent the larger sample of women who participated in the TRIO Study. The qualitative sample participants were not selected based on perceptions of being co-designers. It is also worth considering whether women who felt more engaged with the study and who felt respected by the research team were more likely to participate in the qualitative components. Additionally, the study sought views from end-users after two of the products were approved or in late clinical stages for HIV prevention, although none had progressed very far yet for a dual or MPT indication.

Our study has demonstrated that when study participants are engaged to give opinions on the design of health products before they are developed and released into the market, they embrace their role as co-designers if they are treated well by study staff, given a chance to make choices during the study period, are recognized as equal partners of the researchers, and are motivated by a desire to help themselves and others. Studies are needed to engage end-users of multi-purpose technologies as co-designers across the different product development phases.

### Consent

All study participants provided written informed consent in a language that was understandable to them.

## Data availability

### Underlying data

Given the sensitive nature of the qualitative data collected in this study, RTI International is unable to make the dataset publicly available. Those wishing to gain access to the data are invited to contact the corresponding author and will be asked to complete a data use request and execute a Data Use Agreement (DUA) with RTI International before gaining access. Those who have completed a signed DUA will be granted access to the data.

### Extended data

Open Science Framework: TRIO Project, https://doi.org/10.17605/OSF.IO/MYC9R ( Wagner & Shapley-Quinn, 2020).

This project contains the following extended data:

- 2017_May 01 Trio dissemination slides_ka_FO.ppt (dissemination workshop guidance slides)
- Trio_IDI_FGD_Codebook_v1.4_no additional info.docx (Codebook)
- Trio FGD Guide_V2.1.pdf (Focus group discussion guide)
- Trio IDI 1_V1.0.pdf (Round 1 interview guide)
- Trio IDI 2_V2.1.pdf (Round 2 interview guide)
- Trio Male Partner IDI Guide_V2.1.pdf (Male partner interview guide)

Data are available under the terms of the Creative Commons Zero "No rights reserved" data waiver (CC0 1.0 Public domain dedication).
