# Supplementary material for: “I felt like a TRIO champion”: end-user perspectives on their role as co-designers of multi-purpose technologies
Source: Gates Open Res. 2020 Oct 26;4:163. [Version 1] doi: 10.12688/gatesopenres.13182.1 (PMC8028846; doi:10.12688/gatesopenres.13182.1)
Supplement: Peer review history

# Peer review: Reviewer response for version 1

**DOI:** 10.21956/gatesopenres.14386.r30511

**Published:** 2021-04-06

**Authors:** Sarah E. Asuquo

**Affiliations:** 1 UNC Gillings School of Global Public Health, Chapel Hill, NC, USA

**Methods:**

- More detail is needed in describing the translation process. It is not mentioned who was involved in translation, their proficiency in the languages translated and in the English language, how the English translation was verified, and if reverse translation was done or not.

**Table 2:**

- Having "Total (n=165) and total for each site written at the top of the table is distracting when the numbers don't add up. Consider removing or making the footnote clearer. The footnote mentioned how many women participated in more than one qualitative activity but did not mention for which site. 

**Discussion:**

- "The authors reported that only 7 of 49 studies included people with dementia in multiple phases of development and iteratively consulted them across all the four co-designer phases while, similar to our study, the majority engaged end-users in the development (38/49) and generative (25/49) phases only." -  The sentence is winding and unclear. Consider revising.
- "As experts of their own lives, end-users should be offered an opportunity to influence the design processes of products being development for them..." - Wrong grammar. Change "being development" to "developed"

Is the work clearly and accurately presented and does it cite the current literature?

Yes

If applicable, is the statistical analysis and its interpretation appropriate?

Not applicable

Are all the source data underlying the results available to ensure full reproducibility?

No source data required

Is the study design appropriate and is the work technically sound?

Yes

Are the conclusions drawn adequately supported by the results?

Yes

Are sufficient details of methods and analysis provided to allow replication by others?

Partly

Reviewer Expertise:

My areas of research include qualitative research, community-based participatory research, public health, and health behavior

I confirm that I have read this submission and believe that I have an appropriate level of expertise to confirm that it is of an acceptable scientific standard.

# Peer review: Reviewer response for version 1

**DOI:** 10.21956/gatesopenres.14386.r29880

**Published:** 2020-11-20

**Authors:** Kenneth Ngure

**Affiliations:** 1 Department of Global Health, University of Washington, Seattle, WA, USA; 2 Jomo Kenyatta University of Agriculture and Technology, Juja, Kenya

**Comments to the Author**

 This is an interesting and well written paper focusing on how the young women who participated in the TRIO study in Kenya and South Africa perceived themselves as co-designers in the development of the three MPT products. I am recommending minor changes because I think there are a few details especially in the methods that would strengthen the paper.

**Methods**

 This is a well written manuscript. The investigators will need to provide a rationale of using the three qualitative methods - IDIs, FGDs and dissemination workshops. Also what informed the number of IDIs, FGDs, and dissemination workshops especially the IDIs - was saturation achieved?

 It will be good to mention the proportion of women who participated in more than one research activity e.g. how many participated in IDIs only, IDIs and FGDs, or all the three activities?

 Specify the number of interviewers, training, and gender to enable the reader put the findings in context. Also good to include the mean duration of the IDIs and FGDs plus the average number of participants per FGD.

 The analysts could also be identified by initials since if they are co-authors.

 Include more details of how the thematic analysis was conducted and provide relevant references where readers can refer to understand the authors analytical approach better.

**Results**

 Although the quotes are identified by interview type, I would recommend an addition of more identification for IDIs (e.g. age, marital status of the participants, etc.) because some of these attributes could influence views.

 It would be great to highlight the other non-key (minor) themes, even if briefly.

**Discussion**

 Could the researchers push their analysis a bit more and reflect on how these findings of these three methods agree or differ and therefore discuss surrounding points of convergence and divergence between the three groups interviewed?

 Further discussion of study limitations is warranted, including how the context being the larger TRIO study was likely to affect the findings and their generalizability.

Is the work clearly and accurately presented and does it cite the current literature?

Yes

If applicable, is the statistical analysis and its interpretation appropriate?

Not applicable

Are all the source data underlying the results available to ensure full reproducibility?

Yes

Is the study design appropriate and is the work technically sound?

Yes

Are the conclusions drawn adequately supported by the results?

Yes

Are sufficient details of methods and analysis provided to allow replication by others?

Yes

Reviewer Expertise:

HIV prevention, behavioral research, qualitative  and quantitative  research methods

I confirm that I have read this submission and believe that I have an appropriate level of expertise to confirm that it is of an acceptable scientific standard.
